# Early Versus Delayed Mobilization After Rotator Cuff Repair: A Systematic Review of Re-tear Rates, Pain, Function, and Range of Motion

**DOI:** 10.7759/cureus.108328

**Published:** 2026-05-05

**Authors:** Mahmoud M Hassaan, Lama M Barnawi, Abdulmalik M Almukhashi, Ali A Alshehri, Abdullah K Alnajjar, Zahraa A Alnakhli, Mhmud I Almslmani, Layan F Alanazi, Fahad M Almulhim, Mohammed A Alanazi, Enad S Almalki

**Affiliations:** 1 College of Medicine, Jazan University, Jazan, SAU; 2 Faculty of Medicine, Tabuk University, Tabuk, SAU; 3 Faculty of Medicine, King Faisal University, Al Ahsa, SAU; 4 Orthopedic Surgery, King Salman Armed Forces Hospital, Tabuk, SAU

**Keywords:** delayed mobilization, early mobilization, functional outcomes, pain outcomes, postoperative rehabilitation, rehabilitation protocol, re-tear rate, rotator cuff repair, shoulder range of motion, systematic review

## Abstract

Rotator cuff tears are among the most common musculoskeletal conditions, with surgical repair rates increasing substantially over recent decades. Despite advances in surgical technique, re-tear rates remain a significant concern, particularly for large and massive tears. Postoperative rehabilitation is a critical determinant of both tendon healing and functional recovery, yet there is no consensus on the optimal timing of active mobilization, with the central debate focusing on whether early motion reduces stiffness at the expense of higher re-tear rates or whether delayed immobilization provides superior tendon healing without compromising long-term function. A systematic search of PubMed (MEDLINE), the Cochrane Library (CENTRAL), and Google Scholar was conducted in February 2026, and studies comparing early active or passive mobilization (initiated within 1-4 weeks) with delayed mobilization (≥4-6 weeks of immobilization) after rotator cuff repair were included. Outcomes assessed included re-tear rates, pain measured using the visual analog scale (VAS), functional scores including the American Shoulder and Elbow Surgeons (ASES) score, Constant score, University of California, Los Angeles (UCLA) Shoulder Rating Scale, Western Ontario Rotator Cuff (WORC) index, and Disabilities of the Arm, Shoulder and Hand (DASH) questionnaire, as well as range of motion. Risk of bias was assessed using the Cochrane Risk of Bias 2 tool for randomized controlled trials (RCTs), and the Newcastle-Ottawa Scale (NOS) for cohort studies, and certainty of evidence was evaluated narratively using the Grading of Recommendations Assessment, Development and Evaluation (GRADE) framework. Twelve studies (10 RCTs and 2 cohort studies; total 1,255 patients) met the inclusion criteria. Nine studies reporting re-tear rates found no statistically significant difference between early and delayed mobilization groups. Pain outcomes were comparable in most studies, although two trials reported lower pain scores in the early mobilization group. Functional outcomes showed no significant long-term differences in the majority of studies, though several reported short-term advantages with early mobilization. Early mobilization consistently improved range of motion in the short term (6 weeks to 3 months), particularly forward flexion and external rotation, but these advantages generally diminished by 6-12 months. Overall, early active mobilization after rotator cuff repair does not appear to increase re-tear rates and provides short-term range of motion benefits compared with delayed mobilization, while long-term functional outcomes remain comparable between protocols, although caution may be warranted in patients with large tears and advanced fatty infiltration, supporting an individualized rehabilitation approach based on tear size and tissue quality.

## Introduction and background

Rotator cuff tears are among the most common musculoskeletal shoulder disorders, affecting both working-age and older adults. Surgical repair, specifically rotator cuff repair (RCR), has increased substantially over recent decades as indications for operative treatment have expanded [1-5]. The prevalence of full-thickness tears increases with age, and many lesions progress over time, supporting the role of timely surgical management. Despite advances in arthroscopic and open techniques, achieving durable tendon-to-bone healing remains challenging, particularly in large and massive tears where re-tear rates remain high [1,4,6].

Postoperative rehabilitation following RCR plays a critical role in outcomes. Rehabilitation protocols aim to balance two competing goals: protecting the repair to allow biological healing while restoring shoulder mobility and function. Traditional approaches often involve immobilization for approximately four to six weeks to minimize mechanical stress on the repair [5-10]. This strategy is based on the concern that early loading may exceed the initial strength of the healing tendon and increase the risk of structural failure.

In contrast, early mobilization protocols advocate for initiating controlled shoulder movement soon after surgery to reduce stiffness, prevent adhesive capsulitis, and facilitate earlier functional recovery. However, evidence comparing early and delayed mobilization remains inconsistent due to variability in study designs, tear characteristics, and outcome measures [1-12].

The objective of this systematic review is to evaluate the current evidence comparing early active mobilization with delayed mobilization following RCR, with respect to re-tear rates, pain, range of motion, and functional outcomes.

## Review

Methods

Search Strategy

Study selection followed Preferred Reporting Items for Systematic Reviews and Meta-Analyses (PRISMA) guidelines [13]. A systematic literature search was performed in February 2026 in three electronic databases: PubMed (Medical Literature Analysis and Retrieval System Online), the Cochrane Library (Central Register of Controlled Trials), and Google Scholar. Search terms combined “rotator cuff repair” with “early motion,” “early mobilization,” “accelerated rehabilitation,” “delayed,” “immobilization,” and “conservative rehabilitation” using Boolean operators. No date restrictions were applied. Reference lists of included studies and relevant reviews were also manually screened to identify additional eligible studies (Table 1).

**Table 1:** Literature search strategy used for database identification of studies This table summarizes the systematic search strategy used to identify eligible studies for inclusion in the review. It includes the databases searched, key search terms, date of search, applied filters, and additional supplementary search methods such as manual reference screening. The search was conducted in accordance with PRISMA guidelines [13].

Database	Search terms	Date of search	Filters applied	Additional search methods
PubMed (MEDLINE)	rotator cuff repair AND (early mobilization OR early motion OR accelerated rehabilitation OR delayed immobilization)	46054	None	Manual reference screening
Cochrane Library (CENTRAL)	same keyword strategy	46054	None	Hand-search of reviews
Google Scholar	same keyword strategy	46054	None	Screening of citations

Eligibility Criteria

Eligibility criteria were defined using the Population, Intervention, Comparison, and Outcomes (PICO) framework [14]. The population included adults undergoing surgical rotator cuff repair using arthroscopic, mini-open, or open techniques. The intervention was early mobilization, defined as initiation of active or passive shoulder movement within one to four weeks postoperatively. The comparator was delayed mobilization, defined as immobilization for at least four to six weeks before starting active range of motion exercises. Outcomes included re-tear rates assessed by imaging, pain measured using the visual analog scale (VAS) [15], functional outcomes (American Shoulder and Elbow Surgeons score [16], Constant-Murley score [17], University of California Los Angeles score [18], Western Ontario Rotator Cuff index [19], and Disabilities of the Arm, Shoulder and Hand score [20]), and shoulder range of motion (ROM). Eligible study designs included randomized controlled trials (RCTs) and prospective or retrospective comparative cohort studies. Case reports, case series, animal or cadaver studies, reviews, meta-analyses, editorials, and protocols were excluded (Table 2).

**Table 2:** Inclusion and exclusion criteria for study selection This table outlines the predefined eligibility criteria used to determine study inclusion in this systematic review. Criteria are structured according to population, intervention, comparison, outcomes, study design, and language. Each category includes both inclusion and exclusion conditions applied during screening based on the PICO framework [14]. VAS: visual analog scale; ROM: range of motion; ASES: American Shoulder and Elbow Surgeons score; UCLA: University of California, Los Angeles shoulder rating scale; WORC: Western Ontario Rotator Cuff index; DASH: Disabilities of the Arm, Shoulder and Hand questionnaire; PICO: Population, Intervention, Comparison, and Outcomes

Criterion category	Inclusion criteria	Exclusion criteria
Population	Adults undergoing rotator cuff repair (arthroscopic, mini-open, or open)	Pediatric patients, animal or cadaver studies
Intervention	Early mobilization (active or passive within 1–4 weeks post-op)	No defined early mobilization protocol
Comparison	Delayed mobilization (immobilization ≥4–6 weeks)	No comparator group
Outcomes	Re-tear rates, VAS pain, ROM, ASES, Constant, UCLA, WORC, DASH	Studies not reporting any of these outcomes
Study design	RCTs and comparative cohort studies	Case reports, case series, reviews, meta-analyses, editorials, protocols
Language	English	Non-English studies

Study Selection

The search yielded 501 records (PubMed: Medical Literature Analysis and Retrieval System Online: 312, Cochrane Library: 89, Google Scholar: 100). After removing 147 duplicates, 354 records were screened by title and abstract. A total of 296 studies were excluded due to irrelevant population or intervention, inappropriate study design, non-English language, pediatric population, animal or cadaver models, or being reviews, editorials, or case reports. Fifty-eight full-text articles were assessed for eligibility, of which 46 were excluded for reasons including being systematic reviews or meta-analyses, absence of a comparator group, incorrect outcomes, case series design, lack of comparison between early and delayed mobilization, or ongoing study protocols. Ultimately, 12 studies met the inclusion criteria. Screening was performed by a single reviewer with verification, which represents a methodological limitation.

Data Extraction

Data were extracted using a standardized form. Extracted variables included author, year, country, study design, sample size, participant characteristics, details of early and delayed mobilization protocols, outcome measures, follow-up duration, and key findings. Data extraction was completed by a single reviewer and verified against the original articles.

Quality Appraisal and Risk-of-Bias Assessment

Methodological quality and risk of bias were assessed using design-specific tools. Randomized controlled trials were evaluated using the Cochrane Risk of Bias 2 tool, which assesses bias from randomization, deviations from intended interventions, missing outcome data, outcome measurement, and selective reporting [21]. Non-randomized cohort studies were assessed using the Newcastle-Ottawa Scale, which evaluates selection, comparability, and outcome assessment domains [22]. Overall study quality was classified as high, moderate, or low based on scoring thresholds. Certainty of evidence for each outcome was assessed narratively using the Grading of Recommendations, Assessment, Development, and Evaluations (GRADE) approach [23].

Data Synthesis

A meta-analysis was not performed due to heterogeneity in study design, interventions, outcome measures, and follow-up durations. A narrative synthesis was conducted following Synthesis Without Meta-analysis guidelines. Studies were grouped by outcome domain (re-tear rates, pain, function, and range of motion), and results were compared qualitatively with consideration of effect direction, consistency across studies, and study quality.

Results

Study Selection

**Figure 1:**
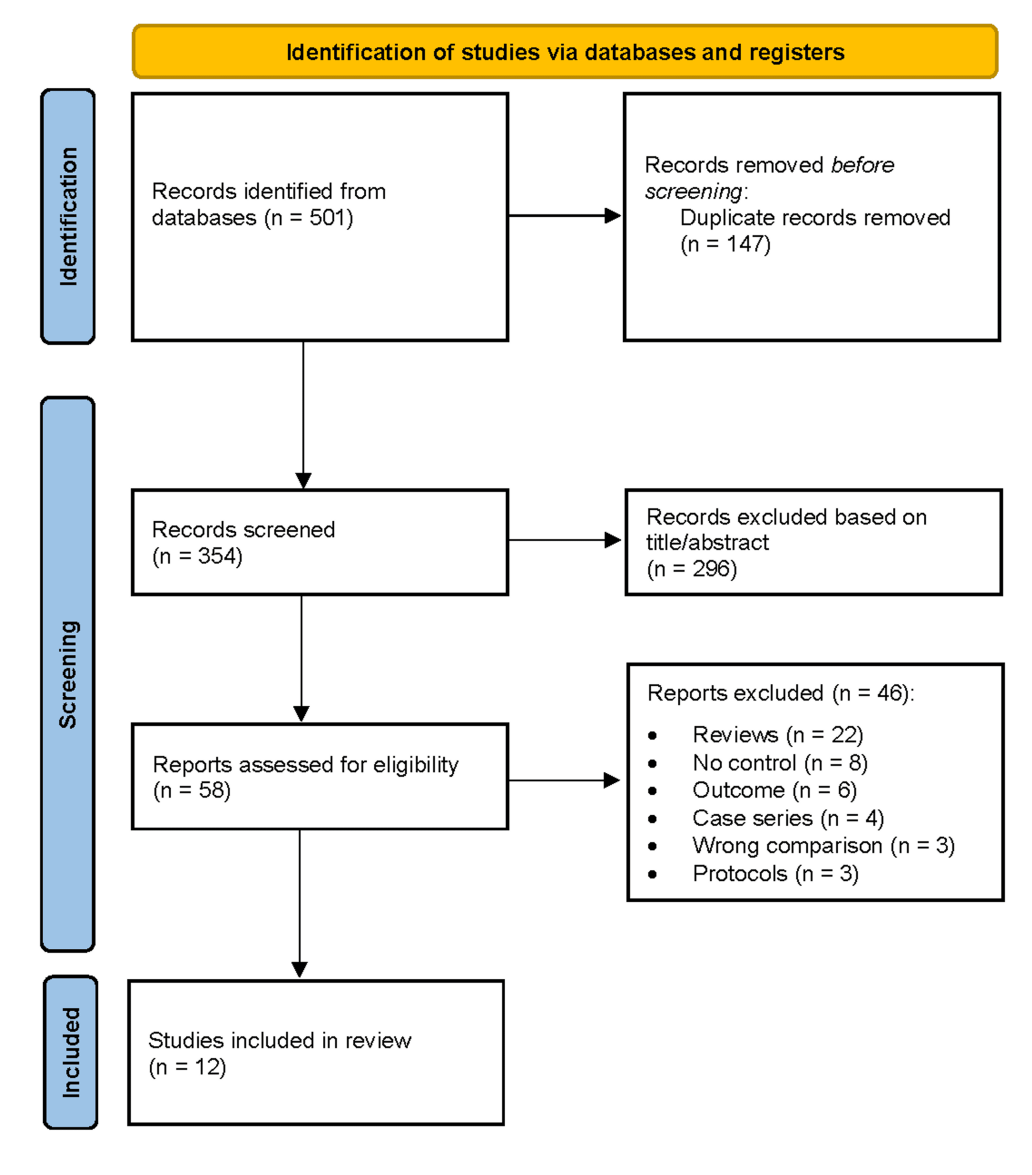
PRISMA flow diagram depicting the study selection process for the systematic review PRISMA (Preferred Reporting Items for Systematic Reviews and Meta-Analyses) flow diagram detailing the study selection process for this systematic review [13]. Following the identification of records through database searching and other sources, duplicates were removed, and the remaining records were screened. Full-text articles were assessed for eligibility, with exclusions documented along with reasons. Studies meeting the inclusion criteria were included in the final synthesis.

The systematic search identified 501 records from PubMed (Medical Literature Analysis and Retrieval System Online), the Cochrane Library (Central Register of Controlled Trials), and Google Scholar. After removal of 147 duplicates, 354 records were screened by title and abstract, and 296 were excluded. Fifty-eight full-text articles were assessed for eligibility, with 46 excluded due to inappropriate study design, lack of comparator, incorrect outcomes, or irrelevant populations. Twelve studies met the inclusion criteria and were included in the final analysis (Figure 1).

Study Characteristics

The 12 included studies comprised 10 randomized controlled trials (RCTs) [1-10] and 2 comparative cohort studies (1 prospective and 1 retrospective) [11,12]. Studies were conducted between 2012 and 2025 across Canada, Austria, the United States, South Korea, France, Iran, China, and Italy. A total of 1,255 participants were included, with sample sizes ranging from 30 to 206 and mean ages from approximately 45 to 63 years. Follow-up ranged from six months to four years. Most studies included patients with small to medium full-thickness rotator cuff tears treated arthroscopically (Table 3).

**Table 3:** Characteristics and findings of included studies This table summarizes the key characteristics and findings of the 12 studies included in this systematic review, comparing early versus delayed mobilization following rotator cuff repair. It includes study design, country, sample size, patient population, rehabilitation protocols (early and delayed), outcome measures, and principal findings related to re-tear rates, pain, range of motion, and functional outcomes. Studies comprise randomized controlled trials and cohort studies with varying follow-up durations and rehabilitation protocols. ASES: American Shoulder and Elbow Surgeons score; CMS: Constant-Murley score; CPM: continuous passive motion; DASH = Disabilities of the Arm, Shoulder and Hand questionnaire; ER: external rotation; HRQOL: health-related quality of life; MRI: magnetic resonance imaging; N: sample size; OSS: Oxford Shoulder Score; POD: postoperative day; ROM: range of motion; RC: rotator cuff; RCT: randomized controlled trial; SST: Simple Shoulder Test; UCLA: University of California Los Angeles Shoulder Rating Scale; VAS: visual analogue scale; WORC: Western Ontario Rotator Cuff index

Study ID	Country	Study Design	Population	N	Intervention (Early)	Comparison (Delayed)	Key Outcomes	Main Findings
Sheps [1]	Canada	RCT (Level I, multicenter)	Full-thickness rotator cuff tears, arthroscopic repair	206	Active ROM from POD 1, self-weaned from sling for 6 weeks	Sling immobilization for 6 weeks, no active ROM	Re-tear, ROM, pain, HRQOL, strength	Re-tear 30% vs 33% (p > 0.8); better ROM at 6 weeks (p < 0.03); no difference in pain or function
Sheps [2]	Canada	RCT (multicenter)	Full-thickness RC tears (<5 cm), mini-open repair	189	Active ROM from POD 1, sling as needed	Sling for 6 weeks, no active ROM	ROM, pain, function	Improved abduction (p = 0.002) and elevation (p = 0.006) at 6 weeks; no difference at ≥3 months
Raschhofer [3]	Austria	RCT (pilot, observer-blinded)	Post–rotator cuff surgery patients	30	Early isometric loading from the early postoperative period	Primary passive motion only	Constant-Murley score, pain, DASH	Improved Constant-Murley scores at all time points; greater pain reduction at 6 and 24 weeks
Mazzocca [4]	USA	RCT (investigator-blinded)	Single-tendon RC tear, arthroscopic repair	73	Motion starting 2–3 days postoperatively	Motion starting at 28 days postoperatively	WORC, re-tear (MRI)	Re-tear 34% vs 31% (p = 0.78); no WORC difference at 6 months; mixed model favored early mobilization (p = 0.04)
Keener [5]	USA	RCT (Level I)	Full-thickness RC tear <30 mm, arthroscopic double-row repair	124	Pendular exercises immediately; passive ROM from POD 7	Immobilization for 6 weeks, then passive ROM	Re-tear, ROM, ASES, SST, Constant, VAS	Re-tear 9.5% vs 5.7% (p = 0.46); improved ROM at 3 months; no functional difference
Cuff [6]	USA	RCT (Level I)	Full-thickness supraspinatus tear, arthroscopic repair	68	Passive elevation/rotation from POD 2	Immobilization for 6 weeks, pendular exercises only	Re-tear, ASES, ROM, satisfaction	Re-tear 15% vs 8% (p > 0.05); no difference in ASES or ROM at 12 months
Kim [7]	South Korea	RCT (Level I)	Small–medium RC tears, arthroscopic repair	105	Abduction brace + early passive motion 3–4×/day	Abduction brace only; active-assisted after removal	Re-tear, ROM, VAS, Constant, SST, ASES	Re-tear 12% vs 18% (p = 0.429); no significant differences in ROM, pain, or function
Lee [8]	South Korea	RCT (Level II)	Medium–large RC tears, arthroscopic repair	64	Frequent manual therapy and unlimited self-passive stretching	Limited CPM and self-passive exercise	Re-tear (MRI), ROM, UCLA	Re-tear 23.3% vs 8.8% (p = 0.11); faster ROM recovery at 3 months; UCLA better at 3 months (p < 0.01)
Arndt [9]	France	RCT (Level II, multicenter)	Non-retracted supraspinatus tear, arthroscopic repair	92	Passive ROM from POD 2, CPM, pendular exercises	Sling immobilization for 6 weeks, pendular exercises only	Re-tear, Constant, ROM	Re-tear 22.4% vs 16.3% (p = 0.5); Constant score improved by 7.9 points (p = 0.045); better ROM at 3–6 months
Nikpay [10]	Iran	RCT (Level I)	Full-thickness tears, arthroscopic/open repair	120	Passive flexion/abduction within 24 hours	Immobilization for 6 weeks	Re-tear (MRI), VAS, UCLA, stiffness, ROM	Re-tear 3.3% vs 1.7% (p = 0.56); lower VAS (p = 0.02); higher UCLA (p = 0.01)
Tang [11]	China	Prospective cohort	Unilateral arthroscopic RC repair	84	Early active mobility exercises	Passive only weeks 0–6; active weeks 6–12	Re-tear, ROM, VAS, CMS, DASH, SST	Re-tear 8.83% vs 9.25% (p = 0.298); improved ROM at 6 weeks (p < 0.001); no long-term difference
Longo [12]	Italy	Retrospective cohort	Full-thickness RC tears, arthroscopic repair	100	Free passive ROM + pendulums from POD 1	Limited passive flexion (wk 0–2); ER/pendulums from week 2	UCLA, Wolfgang, OSS, ROM, strength	Conservative protocol showed better active forward flexion (p = 0.04); no other differences

Quality Assessment and Risk of Bias

Overall methodological quality was moderate to good. Among RCTs, four had low risk of bias [5-7,9], while six had some concerns [1-4,8,10]. Common limitations included a lack of participant blinding, potential detection bias due to unblinded assessors, and attrition. Both cohort studies were rated as moderate quality (6/9 on the Newcastle-Ottawa Scale) (Table 4) [11,12].

**Table 4:** Risk of bias and methodological quality of included studies This table presents the risk of bias (RoB) and methodological quality assessment of the 12 studies included in this systematic review, comparing early versus delayed mobilization after rotator cuff repair. Randomized controlled trials (RCTs) were assessed using the Cochrane Risk of Bias 2 (RoB 2) tool, with domains summarized as key sources of bias, including randomisation, deviations from intended interventions (performance bias), missing outcome data (attrition bias), outcome measurement (detection bias), and selective reporting [15]. Cohort studies were assessed using the Newcastle–Ottawa Scale (NOS), with domains covering selection bias, comparability, and outcome assessment [16]. Overall study quality was graded as high or moderate based on the RoB 2 domain judgments for RCTs and NOS-derived methodological quality for cohort studies. “Key bias domains/quality notes” provides a condensed narrative summary of the most relevant methodological limitations for each study. NOS: Newcastle-Ottawa Scale; RCT: randomized controlled trial; RoB 2: Cochrane Risk of Bias 2 tool.

Study ID	Study design	Tool	Key bias domains/quality notes	Overall quality
Sheps [1]	RCT	RoB 2	Low risk randomization; high performance bias (no blinding); some concerns in outcome measurement	Moderate
Sheps [2]	RCT	RoB 2	Low risk randomization; high performance bias; some concerns in outcome measurement	Moderate
Raschhofer [3]	RCT (pilot)	RoB 2	Low risk randomization; some concerns due to small sample and lack of blinding	Moderate
Mazzocca [4]	RCT	RoB 2	Low risk randomisation; some attrition and performance bias concerns	Moderate
Keener [5]	RCT	RoB 2	Low risk across all domains	High
Cuff & Pupello [6]	RCT	RoB 2	Low risk across all domains	High
Kim [7]	RCT	RoB 2	Low risk across all domains	High
Lee [8]	RCT	RoB 2	Some concerns in randomization and intervention adherence	Moderate
Arndt [9]	RCT	RoB 2	Low risk randomization; some detection bias concerns	Moderate
Nikpay [10]	RCT	RoB 2	Low risk across all domains	High
Tang [11]	Cohort	NOS	Moderate selection and outcome quality; limited comparability	Moderate
Longo [12]	Cohort	NOS	Moderate selection and outcome quality; limited comparability	Moderate

Re-tear Rates

Nine studies reported re-tear rates [1,4-11]. None demonstrated statistically significant differences between early and delayed mobilization. Reported re-tear rates ranged from 3.3% to 34% in early mobilization groups and 1.7% to 33% in delayed groups. Some studies showed numerically higher re-tear rates with early mobilization, particularly in larger tears, while others showed no difference or a slight advantage for early protocols. Overall, tear size and tendon quality appeared to influence re-tear risk more than rehabilitation timing. Evidence certainty was low to moderate due to imprecision and clinical heterogeneity.

Pain Outcomes

Six studies reported pain using the VAS [1,3,5,7,10,11]. Most found no significant differences between groups. Two studies reported lower pain scores with early mobilization at mid-term follow-up. Overall, early mobilization did not increase postoperative pain and may provide modest short-term pain reduction. Evidence certainty was moderate.

Functional Outcomes

All studies reported functional outcomes using validated measures, including the ASES score, Constant-Murley score, UCLA score, WORC index, and DASH score. Most studies showed no significant long-term differences between groups. Several studies demonstrated short-term functional advantages with early mobilization, which generally resolved by 6-12 months. One long-term study also found slightly better forward flexion in the delayed group. Overall certainty was moderate.

Range of Motion

All studies reported ROM outcomes. Early mobilization consistently resulted in better short-term shoulder motion, particularly forward flexion and external rotation, within the first six weeks to three months. These differences generally disappeared by 6-12 months, with most studies showing convergence between groups over time. Evidence certainty was moderate to high due to consistent findings across studies.

Summary of Evidence Certainty

Certainty of evidence was low to moderate for re-tear rates, moderate for pain and functional outcomes, and moderate to high for ROM. Overall, early mobilization improves early recovery of motion without clear differences in long-term structural or functional outcomes.

Limitations

This review has several limitations. Most included studies were underpowered to detect differences in re-tear rates, which are relatively infrequent outcomes. There was substantial heterogeneity in definitions of early and delayed mobilization, tear size, and surgical techniques, limiting comparability across studies. Blinding of patients and therapists was not feasible in rehabilitation trials, introducing potential performance bias.

Two included studies were non-randomized cohort designs [11,12], which are more susceptible to selection bias and confounding. One study had a very small sample size (n = 30), limiting generalizability. Follow-up durations varied widely (six months to four years), which may have affected the detection of late failures and long-term outcomes. Study selection was performed by a single reviewer with verification rather than independent duplicate screening. Finally, a meta-analysis was not performed due to heterogeneity in study designs and outcome measures.

## Conclusions

Early active mobilization after rotator cuff repair appears to be a safe rehabilitation strategy, particularly for small to medium tears. Overall, it shows similar long-term re-tear rates and functional outcomes compared with delayed mobilization, while offering better short-term recovery of shoulder range of motion. Pain outcomes are generally comparable between protocols.

Although early mobilization may be appropriate for many patients, rehabilitation should be individualized based on tear characteristics, tissue quality, surgical factors, and patient needs. Further high-quality research with standardized protocols and long-term follow-up is needed to strengthen the evidence base.
